# Awareness of Age-Related Change, Future Time Perspective, and Implications for Goal Management in Older Adulthood

**DOI:** 10.1093/geroni/igaa057.1551

**Published:** 2020-12-16

**Authors:** Bethany Wilton-Harding, Tim Windsor

**Affiliations:** 1 Flinders University, Adelaide, Australia; 2 Flinders University, Adelaide, South Australia, Australia

## Abstract

How people manage their goals is central to adaptation across the lifespan. However, little is known about the individual difference characteristics that predict how and why people use different self-regulatory strategies. The present study aimed to investigate associations of perceived age-related gains and losses, and their interaction, as predictors of flexibility in goal management in older adulthood. We also examined whether future time perspective (FTP) mediated the relationship between AARC and goal flexibility, such that awareness of aging impacts perceptions of time remaining, impacting how individuals evaluate and manage their goals. A community-based sample of 408 adults aged between 60 and 88 years was recruited via an internet-based research platform. Participants completed questionnaire measures of AARC-gains, AARC-losses, FTP, goal disengagement, and goal re-engagement. A flexibility index reflecting tendencies toward use of both goal disengagement and re-engagement strategies was also analyzed. Although AARC-losses was associated with lower goal re-engagement and goal flexibility, this association was weaker among those with higher AARC-gains, indicating that AARC-gains may be protective in the relationship between AARC-losses and goal management. Furthermore, the association between AARC and goal management was mediated by FTP. Higher AARC-gains were associated with more open-ended FTP, which was associated with higher goal re-engagement and lower goal disengagement. On the other hand, higher AARC-losses was associated with more restricted FTP, which was associated with lower goal re-engagement, and higher goal disengagement. Results suggest that subjective awareness of aging (particularly awareness of gains), has important implications for goal management in older adulthood.

